# Supplementary material for: Genome-Wide Identification and Characterization of the PHT1 Gene Family and Its Response to Mycorrhizal Symbiosis in Salvia miltiorrhiza under Phosphate Stress
Source: Genes (Basel). 2024 May 6;15(5):589. doi: 10.3390/genes15050589 (PMC11120713; doi:10.3390/genes15050589)
Supplement: Supplementary file 1 [file genes-15-00589-s001.zip › Table S1 RT-qPCR primer sequence.pdf]

Table S1 RT-qPCR Primer Sequence

Gene name	Forward primer（5′ -3′）	Reverse primer（5′ -3′）
SmActin	GGTGCCCTGAGGTCCTGTT	AGGAACCACCGATCCAGACA
SmPHT1	TGTCCCGGCTGAGATATTCC	ATCTGAAACCCAAACGCACC
SmPHT2	CCCGAGAGAGTTGTGGAAGA	AAGGCAATGTCGAGGAGGAA
SmPHT3	CCGAGTACGCCAACAAGAAGACC	ACCAGACCGCCAGTGAGGATG
SmPHT4	CTCCGCAAAGACGCAATTCT	GAGCTTGATGATCGGAGGGA
SmPHT5	TCTGGCGGATTGTCTTGATGGTTG	GCGGGCGGTTTCAGGCATC
SmPHT6	ATCACAAGTGGACCTTCGC	CGTGTAGTTCTGGATCCCGA
SmPHT7	GAATCTCCAAGTGCTCGACG	GTGTAGTAAATGCGGCCGAG
SmPHT8	CCCTACGATCACTGGACGAA	CGTCTTCGTCTTGTCCTTGC
SmPHT9	TGCAGGTGGAGATAGAAGCC	GCGATTAGGGTTTGAGCTCG
