# Supplementary material for: Genome-Wide Identification and Characterization of the PHT1 Gene Family and Its Response to Mycorrhizal Symbiosis in Salvia miltiorrhiza under Phosphate Stress
Source: Genes (Basel). 2024 May 6;15(5):589. doi: 10.3390/genes15050589 (PMC11120713; doi:10.3390/genes15050589)
Supplement: Supplementary file 1 [file genes-15-00589-s001.zip › Table S2 Protein sequences of PHT1 in Salvia miltiorrhiza, Arabidopsis thaliana, and Oryza sativus.pdf]

**Table S2** Protein sequences of PHT1 in *Salvia miltiorrhiza*, *Arabidopsis thaliana*, and *Oryza sativus*

***Salvia miltiorrhiza***

>SmPHT1;1

MAGDNNLKVLNALDMAKTQWYHFTAIIIAGMGFFTDAYDLFCISLVTKMLGR
IYYHVEGSPKPGSLPPNVSAAVNGVALCGTLAGQLFFGWLGDKLGRKKVYGL
TLMVMCICSIASGLSFGRDAKAVMATLCFFRFWLGFGIGGDYPLSATIMSEYA
NKKTRGAFIAAVFAMQGFGILAGGIFAMTLSAIFDATFKSPPYEVDPSASTIPQA
DYLWRIVLMVGALPALLTFYWRLKMPETARYTALVAKDAKRAAADMAKVL
QADIEAEQHKAEAMASRQEGYDLFSKEFLHRHGSHLLGTTSTWFLLDIAFYS
QNLFQKDIFTAIGWIPPAKTMNAIHEVYTISRAQTLIALCSTVPGYWFTVALID
VIGRFTIQLGGFFFMTVFMFALAIPYNHWTQPEHRIGFVVMYSLTFFFANFGPN
ATTFVVPAEIFPARFRSTCHGISAASGKLGAIVGAFGFQILAQSQDPKKVDVGY
HPGIGVKNSLIVLGVVNLLGLLFTFLVPEAKGKSLEDLSGENEEATD

>SmPHT1;2

MAKKQLEVLNALDVAKTQWYHFTAIVIAGMGFFTDAYDLFCISLVTKLLGRL
YYTVEGAEKPGSLPPNVAAAVNGVAFCGTLTGQLFFGWLGDKLGRKKVYGM
TLMMMVICSIASGLSLGSRPKAVMATLCFFRFWLGFGIGGDYPLSATIMSEYA
NKKTRGAFIAAVFAMQGFGILAGGMVAIIMSSAFKAAYPAPAYEVDPAASLPA
ASDYVWRLIVMFGALPAALTYYWRMKMPETARYTALVAKNAKQAAADMSR
VLQVDLEAEPERVVEEKGKQFGLFSKEFLRRHGLHLLGTTSTWFLLDIAFYSQ
NLFQKDIFSAIGWIPAAKTMNALEEVFRIARAQTLIALCSTVPGYWFTVVFIDR
IGRFAIQIMGFTMMTIFMFALAIPYDHWTHKDNRIGFVIMYSLTFFFANFGPNA
TTFVVPAEIFPARFRSTCHGISSAAGKAGAMVGAFGFLYAAQSKDPKKTDAGY
PAGIGVRNSLLVLGCVNALGVMFTFLVPESNGKSLEEMSRENENENENENVE
MGDVRDARTVPL

>SmPHT1;3

MASSGGSVLATLDSSKTQWYHYKAVIVSGMGFFTDAYDLFCISLLTKLLGRIY
YHVPSSPKPGVLPPSVAAAVNGVALCGTLLGQLFFGLLGDRLGRKKVYGLTLV
IMAFCSIASGLSFGSSPKSVMATLCFFRFWLGFGIGGDYPLSATIMAEYANKKT
RGSFIAAVFAMQGFGILTGGLVGMAVSAAFRVCVAAPPYSVDPVGSTPPEADY
VWRIVLMLGALPAVLSYYWRMKLPETARYTALVAKDTEKACRDMEKVLKVG
IEAAAPESESESDKNSFGLISKEFLWRHGVHLIGTASAWFLIDITYYSQNLFQKE
IFTAVGWLPAAKTMSALDEVFKISLAQFLIALCGTVPGYWATVALIDRIGRFWI
QAQGFLLMTVFMVALAVPYHHWTLNKAGFLVMYALTFFFANFGPNSTTFIVA
AEIFPARLRSTCHGISAAAGKAGAIVGAFGFLYAAQNQDGRKTDAGYPSGIGIR
NSLFILAGINMLGLLATFLVPEPKGRSLEDISQEQQRHQESQV

>SmPHT1;4

MALKVLSALDSAKTQFYHFKAIIVAGMGLFTDAYDLFCLPPIIKLLGRIYYPNR
EVPAAVSSAMVAAALLGAVAGNLVFGHLGDRIGRRRVYGLSLLLMVLSSVSC
GFSACTSRSCVLLTLGIFRFALGVGIGGDYPLSATIMSEFANRRTRGSFIAAVFS
MQGFGILVSSTVTMAVCAAFDSATRAPPDGETPLAADLAWRLILVFAAVPAAL
TYYWRMMMPETARYTALVERNVEQAVRDMQRVLAVSLSQIAEEEETLSPNTA
APSRPKPYPLLSCEFLRRHGRDLFACAAAWFLVDVVFYSSNLFQSKIYGRYLP

KAPMNAFQEAFHVAKLQAIIAVSSTIPGYYAAVYFIDRAGRVKIQMSGFFFMAL
GLLAIGIPYQKYWRNTTNMGFMILYSLTFFFANFGPNTTTFIVPAELFPARFRST
CHGISGAAGKVGAIVGSVGFLWASSSGGGEGMTAALVILGGVCVAGMVVTYL
FTPETMGRSLEENESEDDINNAPCIPSQSSAIANSN
>SmPHT1;5
MADDKNLKVLNALDMAKTQWYHFTAIIIAGMGFFTDAYDLFCISLVTKMLGR
IYYYVEGSPKPGSLPPNVSAAVNGVALCGTLAGQLFFGWLGDKMGRKKVYG
LTLMVMCICSIGSGLSFGRDAKGVMTTLCFFRFWLGFGIGGDYPLSATIMSEY
ANKKTRGAFIAAVFAMQGFGILGGGIFAMMLSAIFDAAFKSPPYEVDPAASTIP
QADYLWRIVLMVGALPALLTFYWRLKMPETARYTALVAKDAKRAAADMAK
VLQADIEAEQHKAEAMASRHASYSLFSKEFLRRHGSHLLGTTSTWFLLDIAFY
SQNLFQKDIFTAIGWIPPAKTMNAINEVYTIGRAQTLIAICSTVPGYWFTVALID
VIGRFTIQLGGFFFMTVFMFALAIPYDHWTQPEHRIGFVVMYSLTFFFANFGPN
ATTFVVPAEIFPARFRSTCHGISAASGKLGAIVGAFGFQLLAQSQDPKKVDAGY
HPGIGVKNSLIALGVVNLLGLLFTFLLPEAKGKSLEDLSGENEEATD
>SmPHT1;6
MASNSISVLNALDNARTQWYHVTAIVIAGMGFFTDAYDLFCISTVSKLLGRLY
YYDPTTGKPGKLPHRVNNMVIGVALVGTLTGQLVFGYLGDKLGRKKVYGITL
ILMASCAICSGLSFGYSPKAVMGTLCFFRFWLGFGIGGDYPLSATIMSEYANKK
TRGAFIAAVFAMQGVGIIFAGLVSMIISRVFLGMYAGPAFSQEHVFSTEPEADH
VWRIVLMIGALPAVLTFYWRMKMPETGRYTALIEGNAKQAAADMGRVLDIEI
NAEQEKIAQFNSANEYKLFSSEFFARHGKHLIGTATTWFLLDIAFYSQNLTQKD
IFPVMGLTQDPTKVSALREMFETSRAMFVIALFGTFPGYWFTVAFIEKIGRFYI
QLVGFFMMSVFMFIIGVKYDYLKDKDHKWTFAALYGLTFFFANFGPNSTTFV
LPAELFPTRVRSTCHALSAASGKAGAMIGAFGIQNYTQDGKVKEIRKAMIILAI
TNLMGFAFTFLLTETKGRSLEEISGEDAGCGDAGWGEERPDNKEDASRNG
>SmPHT1;7
MGSRENLQVLDALDTAKTQLYHFTTIVIAGMGFFTDAYDLFSISLITKLLGRIY
YTTNLNAPKPGTLPPKISSAVTGVALVGTLAGQLFFGWLGDKMGRKKVYGLT
LILMIVCSLASGLSFGKSPKGVMATLCFFRFWLGFGIGGDYPLSATIMSEYANK
KTRGAFIAAVFAMQGFGILTSGIVALAVSAAFDHAYSAPSYAADPARSTVDQA
DYIWRIIVMFGALPAALTFYWRMKMPETARYTALVARNAKQAARDMARVLN
VELAAEEEKVERYADRTSNSFGLFSKEFLRRHGMHLLGTTSTWFLLDIAFYSN
NLFQKDIFTAVGWIPKPETMNAVHEVFRVARAQTLVALFATVPGYWFTVIFIDII
GRFAIQIMGFFFMTVFMFALAIPYDHWTHKENRIGFVVMYSLTFFFANFGPNA
TTFVVPAEIFPARLRSTCHGISAAAGKAGAIIGAFGFLYAAQPKEESKRDKGYP
AGIGIKNSLIVLGCVNFLGLLFTFLVPEAKGKSLEEMSGENETDDYEQTAEGD
ASKGTPPPPA
>SmPHT1;8
MAREQLQVLNALDVAKTQLYHFTAIVVAGMGFFTDAYDLFSISLIAKLLGRIY
YTVPGALKPGTLPPTVSSSVTGVALVGTLSGQLFFGWLGDKMGRKKVYGMT
LLIMIVCSIASGLSFGNSPKGVMTTLCFFRFWLGFGIGGDYPLSATIMAEYANK
KTRGAFVAAVFAMQGFGILFGGIISLIVSAAFDHAYKSPTYERDAAASTPPQAD
YVWRIILMFGALPAALTFYWRMKMPETARYTALVAKNAKQAAQDMGRVLNV

ELEVEEEKVERQQKGNNFGLLSKEFLKRHGLHLFGTTSTWFLLDIAFYSQNLF
QKDVFSAIGWIPPAKTMNAIGEVYKISKAQTLLALCSTVPGYWFTVFFIDIIGR
FAIQMMGFFFMTVFMFAVAIPYDHWTKKDNRIGFVVMYGLTFFFANFGPNATT
FVVPAEIFPARLRSTCHGISAAAGKAGAIVGAYGFLYAAQSKDKTKTDHGFPP
GIGVKNALIVLGVINFLGMLCTLAVPEAKGKSLEEASQENIEESNEQGA
>SmPHT1;9
MARQQMQVLTALDSAKTQWYHFTAIVIAGMGFFTDAYDLFCISLVTKLLGRI
YYHRDGAANPGTLPPNVSSAVNGVAFCGTLAGQLFFGWLGDKLGRKRVYGM
TLMLMIISSAASGLSFGATPKAVMATLCFSRFWLGFGIGGDYPLSATIMSEYAN
KKTRGAFVAAVFAMQGFGILTGGMVAIAVAAAFEAAFPAPAYEHGGGASTVPE
ADYAWRVILMFGALPAALTYYWRAKMPETARYTALVARNAKRAAADMSKVL
QVEIEAEPEKMNDSTNAFGLFSKQFLRRHGLHLLGTTTTWFLLDIAFYSQNLF
QKDIFTAIGWTPPPKTMNAMEEMYRIARAQTLIALCSTVPGYWFTVVFIDRIG
RFAIQFMGFFFMTAFMFALAIPYDHWTRSENRIGFVIMYSLTFFFANFGPNATT
FVVPAEIFPARLRATCHGISAAAGKVGAMVGAFGFLYAAQPREAGKAEAGYP
AGMGVRNALMVLGCVNALGMALTLLVPESKGRSLEEMSMENEEELQREIRT
VPV

***Arabidopsis thaliana***
>AtPHT1;1
MAEQQLGVLKALDVAKTQLYHFTAIVIAGMGFFTDAYDLFCVSLVTKLLGRIY
YFNPESAKPGSLPPHVAAAVNGVALCGTLSGQLFFGWLGDKLGRKKVYGLTL
VMMILCSVASGLSFGHEAKGVMTTLCFFRFWLGFGIGGDYPLSATIMSEYANK
KTRGAFIAAVFAMQGVGILAGGFVALAVSSIFDKKFPAPTYAVNRALSTPPQVD
YIWRIIVMFGALPAALTYYWRMKMPETARYTALVAKNIKQATADMSKVLQTD
IELEERVEDDVKDPKQNYGLFSKEFLRRHGLHLLGTTSTWFLLDIAFYSQNLF
QKDIFSAIGWIPKAATMNATHEVFRIARAQTLIALCSTVPGYWFTVAFIDTIGRF
KIQLNGFFMMTVFMFAIAFPYNHWIKPENRIGFVVMYSLTFFFANFGPNATTFI
VPAEIFPARLRSTCHGISAAAGKAGAIVGAFGFLYAAQSQDKAKVDAGYPPGI
GVKNSLIMLGVLNFIGMLFTFLVPEPKGKSLEELSGEAEVSHDEK
>AtPHT1;2
MAEQQLGVLKALDVAKTQLYHFTAIVIAGMGFFTDAYDLFCVSLVTKLLGRIY
YFNPESAKPGSLPPHVAAAVNGVALCGTLSGQLFFGWLGDKLGRKKVYGLTL
IMMILCSVASGLSFGNEAKGVMTTLCFFRFWLGFGIGGDYPLSATIMSEYANK
KTRGAFIAAVFAMQGVGILAGGFVALAVSSIFDKKFPAPTYAVNRALSTPPQVD
YIWRIIVMFGALPAALTYYWRMKMPETARYTALVAKNIKQATADMSKVLQTD
IELEERVEDDVKDPRQNYGLFSKEFLRRHGLHLLGTTSTWFLLDIAFYSQNLF
QKDIFSAIGWIPKAATMNATHEVFRIARAQTLIALCSTVPGYWFTVAFIDTIGRF
KIQLNGFFMMTVFMFAIAFPYNHWIKPENRIGFVVMYSLTFFFANFGPNATTFI
VPAEIFPARLRSTCHGISAAAGKAGAIIGAFGFLYAAQNQDKAKVDAGYPPGIG
VKNSLIVLGVLNFIGMLFTFLVPEPKGKSLEELSGEAEVSHDEK
>AtPHT1;3
MADQQLGVLKALDVAKTQLYHFTAIVIAGMGFFTDAYDLFCVSLVTKLLGRL
YYFNPTSAKPGSLPPHVAAAVNGVALCGTLAGQLFFGWLGDKLGRKKVYGIT

LIMMILCSVASGLSLGNSAKGVMTTLCFFRFWLGFGIGGDYPLSATIMSEYAN
KKTRGAFIAAVFAMQGVGILAGGFVALAVSSIFDKKFPSPTYEQDRFLSTPPQA
DYIWRIIVMFGALPAALTYYWRMKMPETARYTALVAKNIKQATADMSKVLQT
DLELEERVEDDVKDPKKNYGLFSKEFLRRHGLHLLGTTSTWFLLDIAFYSQNL
FQKDIFSAIGWIPKAATMNAIHEVFKIARAQTLIALCSTVPGYWFTVAFIDIIGR
FAIQLMGFFMMTVFMFAIAFPYNHWILPDNRIGFVVMYSLTFFFANFGPNATTF
IVPAEIFPARLRSTCHGISAATGKAGAIVGAFGFLYAAQPQDKTKTDAGYPPGI
GVKNSLIMLGVINFVGMLFTFLVPEPKGKSLEELSGEAEVDK
>AtPHT1;4
MAREQLQVLNALDVAKTQWYHFTAIIIAGMGFFTDAYDLFCISLVTKLLGRIY
YHVEGAQKPGTLPPNVAAAVNGVAFCGTLAGQLFFGWLGDKLGRKKVYGM
TLMVMVLCSIASGLSFGHEPKAVMATLCFFRFWLGFGIGGDYPLSATIMSEYA
NKKTRGAFVSAVFAMQGFGIMAGGIFAIIISSAFEAKFPSPAYADDALGSTIPQA
DLVWRIILMAGAIPAAMTYYSRSKMPETARYTALVAKDAKQAASDMSKVLQV
EIEPEQQKLEEISKEKSKAFGLFSKEFMSRHGLHLLGTTSTWFLLDIAFYSQNL
FQKDIFSAIGWIPPAQSMNAIQEVFKIARAQTLIALCSTVPGYWFTVAFIDVIGR
FAIQMMGFFFMTVFMFALAIPYNHWTHKENRIGFVIMYSLTFFFANFGPNATT
FVVPAEIFPARFRSTCHGISAASGKLGAMVGAFGFLYLAQNPDKDKTDAGYPP
GIGVRNSLIVLGVVNFLGILFTFLVPESKGKSLEEMSGENEDNENSNNDSRTVP
IV
>AtPHT1;5
MAKKGKEVLNALDAAKTQMYHFTAIVIAGMGFFTDAYDLFSISLVTKLLGRI
YYHVDSSKKPGTLPPNVAAAVNGVAFCGTLAGQLFFGWLGDKLGRKKVYGI
TLMLMVLCSLGSGLSFGHSANGVMATLCFFRFWLGFGIGGDYPLSATIMSEYA
NKKTRGAFIAAVFAMQGFGILAGGIVSLIVSSTFDHAFKAPTYEVDPVGSTVPQ
ADYVWRIVLMFGAIPALLTYYWRMKMPETARYTALVARNTKQAASDMSKVL
QVDLIAEEEAQSNSNSSNPNFTFGLFTREFARRHGLHLLGTTTTWFLLDIAYYS
SNLFQKDIYTAIGWIPAAETMNAIHEVFTVSKAQTLIALCGTVPGYWFTVAFID
ILGRFFIQLMGFIFMTIFMFALAIPYDHWRHRENRIGFLIMYSLTMFFANFGPNA
TTFVVPAEIFPARLRSTCHGISAASGKAGAIVGAFGFLYAAQSSDSEKTDAGYP
PGIGVRNSLLMLACVNFLGIVFTLLVPESKGKSLEEISREDEEQSGGDTVVEMT
VANSGRKVPV
>AtPHT1;6
MANEEQGSILKALDVAKTQWYHVTAVVVSGMGFFTDSYDLFVISLITKLLGRI
YYQVPGSSSPGSLPDGISAAVSGVAFAGTFIGQIFFGCLGDKLGRKRVYGLTLLI
MTICSICSGLSLGRDPKTVMVTLCFFRFWLGFGIGGDYPLSATIMSEYSNKRTR
GAFIAAVFGMQGIGILAAGAVSLLVSAVFESKFPSRAYILDGAASTVPQADYV
WRIILMVGALPALLTYYWRMKMPETARYTALVSKNAEQAALDMTKVLNVDI
EASAAKNDQARVSSDEFGLFSMKFLRRHGLHLLGTASTWFLLDIAFYSQNLF
QKDIFTTIGWLPSAKTMNAIQELYMIAKAQTIIACCSTVPGYFFTVGFIDYMGR
KKIQIMGFAMMTIFMLSLAIPYHHWTLPANRIGFVVLYSFTFFFSNFGPNATTFI
VPAEIFPARIRSTCHGISAASGKAGAMVGSFGFSALVKALGMSNTLYIMAGINL
LGLLLTFTIPETNGKSLEELSGETEPEKIKEKIVV
>AtPHT1;7

MAGDQLNVLNALDVAKTQWYHFTAIIIAGMGFFTDAYDLFCISLVTKLLGRIY
YHVDGSEKPGTLPPNVSAAVNGVAFCGTLAGQLFFGWLGDKLGRKKVYGMT
LMVMVLCSIASGLSFGSNPKTVMTTLCFFRFWLGFGIGGDYPLSATIMSEYAN
KKTRGAFIAAVFAMQGFGILTGGIFAIIVSAAFEAKFPAPTYQIDALASTVPQAD
YVWRIILMVGALPAAMTYYSRSKMPETARYTALVAKDAKLAASNMSKVLQV
EIEAEQQGTEDKSNSFGLFSKEFMKRHGLHLLGTTSTWFLLDIAFYSQNLFQK
DIFSAIGWIPPAQTMNAIQEVFKIARAQTLIALCSTVPGYWFTVAFIDVIGRFAI
QMMGFFFMTVFMFALAIPYDHWTHKENRIGFVAMYSLTFFFANFGPNATTFV
VPAEIFPARFRSTCHGISAASGKLGAMVGAFGFLYLAQSPDKTKTEHGYPPGIG
VKNSLIVLGVVNLLGMVFTLLVPESKGKSLEEMSGENEQNDESSSSSNNNSNN
AVSTA
>AtPHT1;8
MKMTKRLINRYKDPCSVSLIMNSKFRYNKPKGKKQKKKKKALDSESQIKKPT
MPIKVLSSLDVARTQWYHFKAIIVAGMGLFTDAYDLFCIAPVMKMISHVYYN
GDSINTAVLSTSYAIALLGTATGQLVFGYLGDRVGRRRVYGLCLIIMILSSFGCG
FSVCTTRRSCVMVSLGFFRFFLGLGIGGDYPLSATIMSEFANKRTRGAFIAAVF
SMQGLGILVSSAVTMAVCVAFKRSGGGLEVDAAAPTEADLAWRLILMIGALP
AALTFYWRMLMPETARYTALVENNIVQAAKDMQRVMSRSHISDEATTDPPPP
PPPPSYKLFSRCFFRLHGRDLFAASFNWFLVDIVFYTSNLLLSHIFSHYSKKPST
AENVYDAAFEVAELGAIIAACSTIPGYWFTVYFIDKIGRVKIQIMGFFFMAVIYL
VAGIPYSWYWSKHEHNNKGFMVLYGLVFFFCNFGPNTTTFIIPAEHFPARFRST
CHGISGAAGKLGAIVGTVGFLWATKKMESDDKNQIYPEVNRMRIAFLILGGV
CIAGILVTYFFTKETMGRSLEENEHDQDNNAESEDEPQIVDGQSSVSTLLQTR
>AtPHT1;9
MPELSLLSALDAARIQWYHFKAIIVAGMGLFTDAYDLFCIAPIMKMISQIYYH
KDSIGTALLSTSYAIALLGTALGQLIFGYLGDRVGRRKVYGLSLLIMVFSSFGC
GFSVCTTRRSCVMVSLGFFRFVLGLGIGGDYPLSATIMSEFANKRTRGAFIAAV
FSMQGLGILMSSAVTMVVCLAFKNAGEGSSEKTNVAGLETLAPPESDIAWRLI
LMIGALPAALTFYWRMLMPETARYTALVENNVVQAAKDMQRVMSVSMISQI
TEDSSSELEQPPSSSSYKLFSRRFLSLHGRDLFAASANWFLVDVVFYTSNLLLS
QIFNFSNKPLNSTNVYDSAFEVAKLAAIVAACSTIPGYWFTVYFIDKIGRVKIQ
MMGFFLMAVVYLVAGIPYSWYWSKHEKTNKGFMVLYGLIFFFSNFGPNTTTF
IIPAELFPARFRSTCHGISGAAGKFGAIVGTVGFLWATRHHEEDGFPDVKRVRIA
FLILGGVCIAGMIVTYLFTRETMGRSLEENEDEIVSTSAGSSPANELLRRQY

***Oryza sativus***
>OsPHT1;1
MAGGQLNVLSTLDQAKTQWYHFMAIVIAGMGFFTDAYDLFCISLVTKLLGRI
YYTDDSKDTPGALPPNVSAAVTGVALCGTLAGQLFFGWLGDKLGRKSVYGF
TLILMVVCSVASGLSFGSSAKGVVSTLCFFRFWLGFGIGGDYPLSATIMSEYAN
KRTRGAFIAAVFAMQGFGILFGAIVALAVSAGFRHAYPAPSYSDNHAASLVPQA
DYVWRIILMFGTVPAALTYYWRMKMPETARYTALIARNAKQAAADMSKVLH
TQIEESADRAETVAVGGESWGLFSRQFLRRHGLHLLATTSTWFLLDIAFYSQN
LFQKDIFSKVGWIPPAKTMNALEELYRIARAQALIALCGTIPGYWFTVAFIEIM

GRFWIQIMGFAMMTAFMLGLAIPYHHWTTPGHHTGFIVMYGFTFFFANFGPN
STTFIVPAEIYPARLRSTCHGISAAAGKAGAIIGAFGFLYAAQDQHKPEPGYPRG
IGIKNALFVLAGTNFLGTIMTLLVPESKGMSLEVISQEVADGDDEEAAYPK
>OsPHT1;2
MAGSQLNVLVKLDQAKTQWYHFMAIVIAGMGFFTDAYDLFCIALVTKLLGR
LYYTDITKPNPGTLPPNVSSAVTGVALCGTLAGQLFFGWLGDKLGRKSVYGFT
LILMVVCSIASGLSFGHTPKSVIATLCFFRFWLGFGIGGDYPLSATIMSEYASKK
TRGAFIAAVFAMQGFGILFGAIVALVVSAGFRHAYPAPSYAQNPAASLAPQAD
YTWRLILMFGTIPAGLTYYWRMKMPETARYTALVARNAKQAAADMSKVLHA
EIEERPEVVESQVVAGETWGLFSRQFMKRHGMHLLATTSTWFLLDIAFYSQNL
FQKDIFSKVGWIPPAKTMNALEELYRISRAQALIALCGTIPGYWFTVAFIDIVGR
FWIQIMGFFMMTVFMLALGVPYDHWTHPAHHTGFVVLYALTFFFANFGPNST
TFIVPAEIFPARLRSTCHGISAASGKAGAIIGAFGFLYAAQDQHNPDAGYSRGIG
IRNALFVLAGTNFLGMLMTLLVPESKGLSLEEMSKDNVVDETAQEAIAQA
>OsPHT1;3
MADGQLKVLTTLDHARTQWYHFMAIVIAGMGFFTDAYDLFCISLVSKLLGRI
YYTDLAGDNPGSLPPNVSAAVNGVALCGTLAGQLFFGWLGDKLGRKSVYGF
TLVLMVVCSVASGLSFGRTAKGVVATLCFFRFWLGFGIGGDYPLSATIMSEYA
NKRTRGAFIAAVFAMQGFGILFGAIVALVVSAGFRNAYPAPSYADGRAASLVPE
ADYVWRIILMFGTVPAALTYYWRMKMPETARYTALIARNAKQAAADMSKVL
DTEIQEDADRAEAVAAGGAGNEWGLFSRQFVRRHGVHLVATTSTWFLLDIAF
YSQNLFQKDIFSKVGWIPPARTMNAVEEVFRIARAQALIALCGTIPGYWFTVAF
IDVAGRFAIQLMGFAMMTVFMLGLAAPYHHWTTPGNHTGFVVMYGFTFFFA
NFGPNATTFIVPAEIYPARLRSTCHGISAAAGKAGAIVGAFGFLYAAQDPHKPE
AGYKPGIGIRNALFVLAGTNFLGMLMTLLVPESKGMSLEEVSKENVADDEEA
TA
>OsPHT1;4
MAGELKVLNALDSAKTQWYHFTAIVIAGMGFFTDAYDLFSISLVTKLLGRIYY
FNPASKSPGSLPPNVSAAVNGVAFCGTLAGQLFFGWLGDKMGRKKVYGMTL
MLMVICCLASGLSFGSSAKGVMATLCFFRFWLGFGIGGDYPLSATIMSEYANK
RTRGAFIAAVFAMQGFGNLTGGIVAIIVSAAFKSRFDAPAYRDDRTGSTVPQAD
YAWRIVLMFGAIPALLTYYWRMKMPETARYTALVAKNAKQAAADMTQVLNV
EIVEEQEKADEVARREQFGLFSRQFLRRHGRHLLGTTVCWFVLDIAFYSSNLF
QKDIYTAVQWLPKADTMSALEEMFKISRAQTLVALCGTIPGYWFTVFFIDIIGR
FVIQLGGFFFMTAFMLGLAVPYHHWTTPGNHIGFVVMYAFTFFFANFGPNSTT
FIVPAEIFPARLRSTCHGISAAAGKAGAIVGSFGFLYAAQSTDASKTDAGYPPGI
GVRNSLFFLAGCNVIGFFFTFLVPESKGKSLEELSGENEDDDDVPEAPATADHR
TAPAPPA
>OsPHT1;5
MVQDRKVLDALDTAKTQWYHFTAVVIAGMGFFTDAYDLFSISLVTKLLGRIY
YFNPASKSPGSLPPNVSAAVNGVAFCGTLAGQLFFGWLGDKMGRKKVYGMT
LMLMVICCLASGLSFGSSAKGVMATLCFFRFWLGFGIGGDYPLSATIMSEYAN
KRTRGAFIAAVFAMQGFGNLTGGIVAIIVSAAFKLRFDAPAYRDDRAGSTVPQ
ADYAWRIVLMFGAIPALLTYYWRMKMPETARYTALVAKNDKKAAADMARVL

NVELVDEQEKAAAATAAAAEEEAARREQYGLFSREFARRHGHHLLGTTVCW
FVLDIAYYSQNLFQKDIYTAVQWLPKADTMSALEEMFKISRAQTLVALCGTIP
GYWFTVLFIDIVGRFAIQLGGFFLMTAFMLGLAVPYHHWTTPGNHVGFVVMY
AFTFFFANFGPNSTTFIVPAEIFPARLRSTCHGISSAAGKMGAIVGSFGFLYAAQ
STDPSKTDAGYPRGIGVRNSLFLLAGCNVVGFLFTFLVPESKGKSLEELSGENE
MEAEPAAATNSYRQTVPDSGQSE
>OsPHT1;6
MGGGGGEQQQLEVLHALDVAKTQWYHFTAIVVAGMGFFTDAYDLFCISLVT
KLLGRIYYRVDGSPSPGTLPPHVSASVNGVAFVGTLSGQLFFGWLGDKLGRK
RVYGITLMLMVLCSLASALSFGHTPTSVMATLCFFRFWLGFGIGGDYPLSATI
MSEYANKKTRGAFIAAVFAMQGFGIITGGLVAILVSASFRAAFPAPPYGEDPVA
STPPQADFVWRIILMLGALPAALTYYWRTKMPETARYTALVANNAKQAAAD
MSKVLQVVEMRNIGNNGGSRRPFGLFSGEFVRRHGLHLVGTSATWLLLDIAF
YSQNLFQKDIFSAVGWIPKAATMSALEELFRIARAQTLIALCGTVPGYWFTVA
LIDVVGRFKIQAVGFFMMTLFMLTLALPYHHWTAPGKNHVGFLLLYGLTFFFA
NFGPNSTTFIVPAEIFPARLRATCHGISAASGKLGAIVGSFGFLYLAQSPDRSKT
EHGYPPGIGVRNSLFLLAACNLLGLLFTFLVPESKGKSLEEMSGDAEAQEEAP
PPLQTVL
>OsPHT1;7
MAGDQMHVLSALDSAKTQWYHFTAIVIAGMGFFTDAYDLFCISLVTKLIGRV
YYTADGASKPGSLPPNVSAAVNGVAFVGTLTGQLFFGWLGDRVGRKSVYGM
TLLLMIICSVASGLSFGDTPTSVMATLCFFRFWLGFGIGGDYPLSATIMSEYAN
KRTRGAFIAAVFAMQGFGILAGGAVAIGITAIFRSRFPAPPFAADPAASTPPQAD
YVWRLILMFGALPAALTFYWRMRMPETARYTAIVAKNAERAAADMSKVLQV
KITAEQAEMASPVDKPFTSKPFGLFSGEFARRHGFHLLGTTSTWLLLDIAYYSQ
NLFQKDIFSAIGWIPEAKTMSALDELYHIARAQTLIALCGTVPGYWFTVALIDV
VGRFKIQAAGFFVMTAFMLALAVPYDHWTAAGNQIGFVVLYALTFFFANFGP
NATTFIVPAEIYPARLRATCHGISAASGKVGAIVGSFGFLYLAQSPVPAKAAAH
GYPPGIGVRNSLFALAGCSLLGFLLTFLVPEPKGKSLEEMSRENEVGQP
>OsPHT1;8
MARQEQQQHLQVLSALDAAKTQWYHFTAIVVAGMGFFTDAYDLFCISLVTKL
LGRIYYTDLAKENPGSLPPNVAAAVNGVAFCGTLAGQLFFGWLGDKLGRKSV
YGMTLLMMVICSIASGLSFSHTPTSVMATLCFFRFWLGFGIGGDYPLSATIMSE
YANKKTRGAFIAAVFAMQGFGILAGGIVTLIISSAFRAGFPAPAYQDDRAGSTV
RQADYVWRIILMLGAMPALLTYYWRMKMPETARYTALVAKNAKQAAADMS
KVLQVEIQEEQDKLEQMVTRNSSSFGLFSRQFARRHGLHLVGTATTWFLLDIA
FYSQNLFQKDIFTSINWIPKAKTMSALEEVFRIARAQTLIALCGTVPGYWFTVF
LIDIVGRFAIQLLGFFMMTVFMLGLAVPYHHWTTKGNHIGFVVMYAFTFFFAN
FGPNSTTFIVPAEIFPARLRSTCHGISAAAGKAGAIIGSFGFLYAAQDPHKPDAG
YKPGIGVRNSLFVLAGCNLLGFICTFLVPESKGKSLEEMSGEAEDDDDEVAAA
GGGAAVRPQTA
>OsPHT1;9
MAPRIRVLAALDQARTQYYHFKAIVIAGMGLFTDSYDLFCISPVMKIFGRVYY
APSGSVDGSGSGPGVTPPAVVSATVGVALLGAVAGNVVFGALGDRVGRRRVY

GACLLLMVCSSVGSGLSVCRTRRCALASLCFFRFLLGVGVGGDYPLSATIMSE
FANRRTRGAFIAAVFSMQGFGILVSSAVTMAVAAAFDHYTGYPAPLDTPECAD
LAWRIILMAGAVPAALTYYWRMSMPETARYTALVERDVVKATNDIGRVLADL
DLAAVAEEEVAAAALSPPPVTTPPPPRPSYGLFSRRFVRQHGRDLFACAAAWF
LLDIPYYSSTLFQSQIYRPWFPPAAKVNAFQEAFNVAKFQAVIAVASTIPGYFAA
MLLIERAGRRRLQMAGFLLMAVFLFALAGPYDGYWRDHAKTAGYIVLYSLTF
FSANLGPNTTTFILPAELFPARFRSTCHGLSGAAGKLGALVGSIGFLWASQQKD
GAAAGHLPGIGMMYALFVLGGICLLGLALTYAFTPETMTRSLEENESSVQAQS
QVGDGGSDAGNGSDGLRFHELNVLMEAATKSPVSMASSHLSMSPILPHRMSL
>OsPHT1;10
MAPIGVLTALDQARTQYYHFKAIVIAGMGLFTDSYDLFCIAPVMKIVGRVYYS
DGGARPGVTPPAVVSATVGVALLGAVIGNVVFGALGDRVGRRRVYGACLLLM
VCSSVGSGFSVCRTRRCALASLCFFRFLLGVGVGGDYPLSATIMSEFANRRTR
GAFIAAVFSMQGFGILASSAVTMAVAAAFDHYTGYPAPLDTPECADLAWRIIL
MAGAVPAALTYYWRMSMPETARYTALVERDVVKATNDIGRVLADLDLGAVA
EEEVAAALSRPPPPPRPSYGLLSRRFVRQHGRDLFACAAAWFLLDIPYYSSTLF
QSQIYRPLFPAPGLINAFQEAFNVAKFQAVIAVASTIPGYFVAVLLIDRVGRRCL
QMAGFLLMAVFLFALAGPYDGYWRDHGAHAGYIVLYSLTFFSANLGPNTTTF
ILPAELFPARFRSTCHGLSGAAGKLGALVGSIGFLWASQQKDGAAAGHLPGIG
MMYALFVLGGICLLGLALTYVFTPETMMRSLEENESDRAQTQVGDGGSDTEA
AKSPASMASSHLSMSPILPARVSV
>OsPHT1;11
MADADGGSNLAVLDALDSARTQMYHMKAIVIAGMGFFTDAYDLFCISTVSK
LLGRLYYQPDGSTDSKPGALSKTANNMVIGVALVGTLMGQLVFGYFGDKLGR
KRVYGVTLILMAACAIGSGLSFGSSRKAVIGTLCFFRFWLGFGIGGDYPLSATI
MSEYSNKKTRGAFIAAVFAMQGVGIIFAGLVSMIVSSIFLTYNKAPSYKGNHDL
SRQMPAADYVWRIVLMIGAFPALATFYWRMKMPETARYTAIIDGNAKQAAN
DMQKVLSIEIEAEQEKLAKFNAANNYPLLSMEFARRHGLHLIGTTTTWFLLDI
AFYSQNLTQKDIFPAMGLISGAAEVNALTEMFQISKASFLVALLGTFPGYWVT
VALIDKMGRYMIQLIGFFMMSMFMLAMGILYDYLKTHHFLFGLLYALTFFFAN
FGPNSTTFVLPAELFPTRVRSTCHAISAAAGKAGAIVAAFGIQKLTYNSQVKSI
KKALIILSITNMLGFFFTFLVPETMGRSLEEISGEDGNTGAGGGGAPAAANAGV
GVSASDVSRDEKFPASSTEWQTSMHA
>OsPHT1;12
MGRQDQQLQVLNALDAAKTQWYHFTAIIVAGMGFFTDAYDLFCISLVTKLLG
RIYYTDPASPTPGSLPPNIAAAVNGVALCGTLSGQLFFGWLGDKLGRKSVYGM
TLLLMVICSIASGLSFSHTPTSVMATLCFFRFWLGFGIGGDYPLSATIMSEYAN
KKTRGAFIAAVFAMQGFGILAGGVVTLAMSAGFQAAFPAPAYEVNAAASTVP
QADYVWRIILMLGALPAILTYYWRMKMPETARYTALVAKDAKQASSDMAKV
LQVEIEVEEEKLQDITRGRDYGLFSARFAKRHGAHLLGTAATWFLVDVAYYSQ
NLFQKDIFTSIHWIPKARTMSELEEVFRISRAQTLIALCGTVPGYWFTVFLIDIIG
RFKIQLLGFAGMTAFMLGLAIPYHHWTMPGNQVIFVFLYGFTFFFANFGPNAT
TFIVPAEIFPARLRSTCHGISAASGKAGAIIGAFGFLYAAQPQDKAHVDAGYKP
GIGVRNALFVLAGCNLVGFLMTWMLVPESKGKSLEEMSGEADDEEASANGG

ATAVNSSGVEMV
>OsPHT1;13
MAGNQQLRVLHALDIARTQLYHFIAIVIAGMGFFTDAYDLFSISLVADLLGHV
YYHGELPRNIHAAVTGIALCGTVPGQLVFGWLGDKMGRKRVYGITLLLMVVS
SLASGLSFSKHEGMNIIAVLCFFRFWLGVSIGGDYPLSATIMSEYANKRTRGAFI
AAVFAMQGFGNLAAGIIGMIVSAAFKHSSASKIDYAWRIILMFGAIPAALTYH
WRMKMPETARYTALISKNAKKAAKDMSAVLNVNITPDDEVINELARQDEYG
LFSFEFLHRHGLHLLGTTVCWFVLDVTFYSLNIFMKNIFTEVGLLPRLDSEYH
HTLQRMITMTAVHTFISLCGALPGYFFTVAFVDRIGRVKIQLIGFTMMTVFMLC
LAIPYDQWLRHKNKYGFAVMYGLTFFFANFGPNTTTFIIPAEIFPARLRSTCHGI
SGAVGKIGAIVGVFGFLYTEYHIRIFLFVLIGCNLVGFIFTLLLPESKGKSLEDLT
GEIEEFQEEDEGSEVALSRPIHTVPL
