# Supplementary material for: Genome-Wide Identification and Characterization of the PHT1 Gene Family and Its Response to Mycorrhizal Symbiosis in Salvia miltiorrhiza under Phosphate Stress
Source: Genes (Basel). 2024 May 6;15(5):589. doi: 10.3390/genes15050589 (PMC11120713; doi:10.3390/genes15050589)
Supplement: Supplementary file 1 [file genes-15-00589-s001.zip › Table S4 Analysis of cis-elements in SmPHT1 genes.pdf]

Table S4 Analysis of cis-elements in SmPHT1 genes

Function class	Elements	Function	*SmPH1;1*	*SmPH1;2*	*SmPH1;3*	*SmPH1;4*	*SmPH1;5*	*SmPH1;6*	*SmPH1;7*	*SmPH1;8*	*SmPH1;9*
hormone-related	ABRE	ABA-responsiveness	7	7	5	8	6	10	8	8	8
	CGTCA-motif	MeJA-responsiveness	4	6	4	3	2	7	8	3	12
	TGACG-motif	MeJA-responsiveness	4	6	4	3	2	7	8	3	12
	P-box	GA-responsive	0	0	0	0	0	0	0	0	1
	TATC-box	GA-responsive	0	0	1	0	0	0	0	0	0
	TCA-element	SA-responsive	2	1	2	3	2	2	1	4	1
	TGA-element	IAA-responsive	1	1	0	0	0	0	0	1	5
stress-related	GC-motif	anoxic specific inducibility	0	0	1	0	1	3	1	1	2
	LTR	low-temperature responsiveness	3	0	1	2	6	0	1	0	2
	MBS	drought-inducibility	1	1	0	0	0	0	0	1	0
	Sp1	drought-inducibility	2	3	1	4	4	4	1	3	2
	TC-rich repeats	defense and stress responsiveness	0	1	2	0	0	0	0	1	2
	ARE	anaerobic induction	3	1	0	0	2	1	1	3	5
development-related	CAT-box	meristem expression	0	0	0	1	1	0	1	1	0
	GCN4_motif	endosperm expression	0	1	0	0	0	0	1	0	0
	HD-Zip 1	differentiation of the palisade mesophyll cells	0	0	0	0	0	0	0	1	0
	RY-element	seed-specific regulation	0	0	0	0	0	0	1	0	0

	O2-site	zein metabolism regulation	0	0	0	0	1	0	1	4	0
	circadian	circadian control	1	0	1	0	1	2	0	0	0
light-related	ACE	light- responsive	0	0	0	1	0	0	0	0	2
	AE-box	light- responsive	0	1	0	0	1	0	0	1	1
	ATCT-motif	light- responsive	0	0	0	0	0	0	1	1	0
	AT1-motif	light- responsive	0	2	0	0	0	0	0	0	0
	Box4	light- responsive	6	4	4	4	2	3	2	10	3
	Box II	light- responsive	0	0	0	1	0	0	1	0	0
	C-box	light- responsive	0	0	1	0	0	0	0	0	0
	CAG-motif	light- responsive	1	0	0	0	0	0	0	0	0
	G-Box	light- responsive	1	3	2	2	2	3	4	3	1
	G-box	light- responsive	3	4	4	8	4	8	6	5	6
	GA-motif	light- responsive	0	0	0	0	0	0	0	0	2
	GATA-motif	light- responsive	0	0	1	1	0	1	1	2	1
	GT1-motif	light- responsive	1	0	0	1	1	2	2	2	0
	Gap-box	light- responsive	0	0	0	1	0	0	0	0	0
	I-box	light- responsive	0	1	0	0	0	0	1	2	0
	LAMP-element	light- responsive	0	0	0	0	0	0	0	0	1
	MRE	light- responsive	0	0	2	1	0	0	0	0	2
	TCCC-motif	light- responsive	3	1	1	1	2	0	1	2	1
	TCT-motif	light- responsive	1		3	3	1	0	1	1	4
	chs-CMA1a	light- responsive	1	1		1	0	1	0	0	0
	chs-CMA2a	light- responsive	0	0	1	0	0	0	0	0	0
	chs-CMA2b	light- responsive	0	0	0	0	0	1	0	0	0
