# Supplementary figures and images for: Genome-Wide Identification and Characterization of the PHT1 Gene Family and Its Response to Mycorrhizal Symbiosis in Salvia miltiorrhiza under Phosphate Stress
Source: Genes (Basel). 2024 May 6;15(5):589. doi: 10.3390/genes15050589 (PMC11120713; doi:10.3390/genes15050589)

**Table S3** The sequence information for each motif

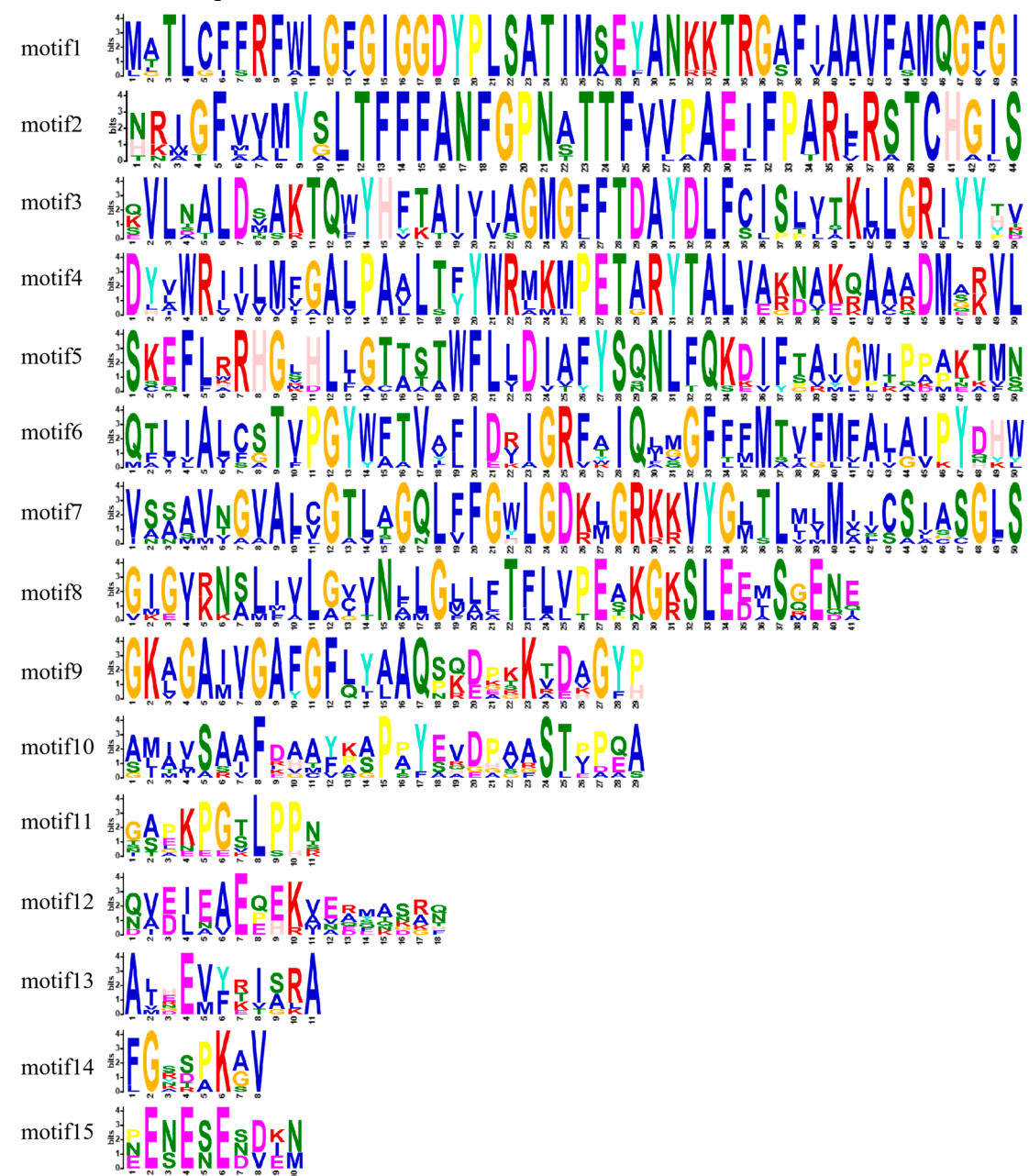

Supplement: Supplementary file 1 [file genes-15-00589-s001.zip › Table S3 The sequence information for each motif.pdf]
